# On the Frequency Carrier Offset and Symbol Timing Estimation for CCSDS 131.2-B-1 High Data-Rate Telemetry Receivers †

**DOI:** 10.3390/s21092915

**Published:** 2021-04-21

**Authors:** Matteo Bertolucci, Riccardo Cassettari, Luca Fanucci

**Affiliations:** 1 Department of Information Engineering, University of Pisa, Via G. Caruso n. 16, 56122 Pisa, Italy; luca.fanucci@unipi.it; 2 IngeniArs S.r.l., Via Ponte a Piglieri n. 8, 56121 Pisa, Italy; riccardo.cassettari@ingeniars.com

**Keywords:** CCSDS 131.2-B-1, timing error detector, TED, frequency estimator, FED, non-data-aided, data-aided, early-late, Gardner, Mueller and Muller, Oerder and Meyr, Lee, multiply and delay, M&D, Kay, Fitz, Luise and Reggiannini, L&R, Mengali and Morelli, M&M, O’Shea et al.

## Abstract

In recent years there have been significant developments in satellite transmitter technology to follow the rapid innovation of sensors on-board new satellites. The CCSDS 131.2-B-1 standard for telemetry downlink, released in 2012, is part of the next generation of standards that aims to support the increased data-rate caused by these improvements in resolution. As a result of its relative novelty, this standard currently lacks in-depth analysis by researchers, but it is also strongly supported by the European Space Agency (ESA) for future missions. For these reasons, it seems important to evaluate how major receiver sub-components, such as timing recovery and carrier frequency correction, can be designed and implemented in new receivers that support this standard. The timing error detectors (TED) and frequency error detectors (FED) were therefore studied on the specific peculiarities of CCSDS 131.2-B-1 in its usual environment of Low Earth Orbit (LEO). Estimators have been evaluated highlighting performances, trade-offs and peculiarities of each one with respect to corresponding architectural choices. Finally, a receiver architecture derived from the paper considerations is proposed in the aim of supporting very different mission scenarios. Specifically, the realized architecture employs a parallel feedforward estimator for the timing recovery section and a novel multi-algorithm feedback frequency correction loop to efficiently cover both low symbol rates (5 Mbaud) and high data-rates (up to 500 Mbaud). This solution represents a good trade-off to support these scenarios in a very compact footprint by pushing the clock frequency to the FPGA limit. The FPGA resources occupation on a Zynq Ultrascale+ RFSoC XCZU28DR FPGA is 5202 LUT, 4851 FF, 5 BRAM, and 21 DSP for the timing recovery part, while the frequency recovery section occupies 1723 LUT, 1511 FF, 2.5 BRAM and 32 DSP.

## 1. Introduction

In recent years, the increase in data transmitted by Earth observation satellites due to improved resolution of on-board instruments has led to an increase in downlink throughput. Given that, due to limitations in the bandwidths dedicated to downlink, more efficient coding and modulation systems have been investigated [1]. For this reason, the Consultative Committee for Space Data Systems (CCSDS) published the CCSDS 131.2-B-1 standard in 2012 [2], which specifies a spectrum-efficient, high data-rate telemetry system based on Serial Concatenated Convolution Codes (SCCC). Moreover, the standard belongs to the class of Variable/Adaptive Coding and Modulation Scheme (VCM/ACM), so the link efficiency can be adaptively optimized during operations. The CCSDS 131.2-B-1 standard features a common frame length for all 27 Modulation and Coding-Rate (ModCod) pairs, with respect to the Physical Layer (PL-Frame) structure. Specifically, it includes 129,600 data symbols; 256 frame marker sequence symbols; and 64 frame descriptor symbols. Compared to DVB-S2, which has a frame marker of only 26 symbols and a frame length that varies from ModCod to ModCod, the difference is quite significant and it is also reflected on many aspects of a receiver’s architecture (e.g., frame synchronization, frequency/phase estimates, etc.). Moreover, the CCSDS 131.2-B-1 also features a higher 64-APSK modulation scheme, whereas the DVB-S2 [3] is limited to the 32-APSK. Due to these differences and the fact that the standard is relatively new without in-depth research so far, but strongly endorsed by the European Space Agency (ESA), it seems important to evaluate the performance of the different timing error detector and frequency estimators for the new generation of receivers employing the CCSDS 131.2-B-1 telemetry downlink standard.

Regarding timing error detection, the investigated schemes are: Early-Late (NDA), Gardner (NDA) [4], Zero-Crossing (DD) [5], Mueller and Muller (DD) [6], Oerder and Meyr (NDA) [7], Lee (NDA) [8]. These algorithms belong to very different classes. Some of these are meant to be implemented in a feedback structure (Early-Late, Gardner, Zero-Crossing, Mueller and Muller), while others are feedforward. From the signal background information requirement, some of the algorithms do not assume any information about the signal (Blind or Non-Data-Aided—NDA), while others are based on symbol-decision (Decision-Directed—DD) made on the received signal or from other sources (e.g., soft-decoder). All these detectors have been evaluated under typical Low Earth Orbit (LEO) scenario conditions, which are the primary target of the standard under analysis.

For the frequency error correction, the selected algorithms belong only to the Data-Aided (DA) class. This remains almost the only possible choice for receivers that need fine frequency estimation in the case of high-modulation constellations (e.g., 32-APSK or 64-APSK). In particular, the Frequency Error Detectors evaluated in this paper are: delay and multiply; Kay [9]; Fitz [10]; L&R [11]; M&M [12]; O’Shea et al. [13]. The algorithms are tested and compared under the normal Additive White Gaussian Noise (AWGN) channel conditions of Earth observation spacecrafts. Details are specifically provided to consider different receiver architectures as the one implementing single feedback loop estimation stage; or the one implementing a fine-coarse approach featuring a feedback loop for coarse estimation and a feedforward loop for fine estimation.

Finally, a CCSDS 131.2-B-1 receiver architectural scheme for data-rates up to 500 Mbaud, derived from the considerations provided in the first analysis part, is presented. The latter receiver employs a feedforward timing estimation with smart considerations related to a two-parallel implementation in the aim of reducing complexity while sustaining high-throughput. Among the analyzed estimators, a multi-algorithm frequency estimator in a single feedback loop fashion is then employed to recover the residual carrier frequency offset. The latter frequency estimator provides a good trade-off in the case of supporting both low symbol-rates (e.g., 5 Mbaud) or high symbol-rates (e.g., 500 Mbaud). These cases provide very different characteristics with respect to impairments. Considering the carrier frequencies for LEO satellites up to Ka-Band ($f_{c}\approx 40$ GHz), a rough Doppler shift estimation can be modeled by $D_{s}=v/c\;\cdot \;f_{c}$ where *v* is the satellite speed, *c* the speed of light and $f_{c}$ the carrier frequency. With this rough estimation, the Doppler effect varies from almost negligible values for the 500 Mbaud (≈0.2%) to considerable high vales in the case of 5 Mbaud (≈20%). The presented architecture and related consideration take all these differences into consideration, providing a good implementation trade-off between the different scenarios.

The reminder of the paper is organized as follows:

- Section 2 presents the different timing error detector algorithms (TED).
- Section 3 presents the different frequency estimation algorithms (FED).
- Section 4 shows TED performances for simulations in relative low earth orbit (LEO) conditions for the considered CCSDS 131.2-B-1 standard.
- Section 5 shows the frequency recovery algorithm performances and trade-offs in the sense of RMS error over the frequency range and different noise levels.
- Section 6 details a possible receiver architecture for the standard under consideration derived from analyses and employing a multi-algorithm FED.
- Section 7 states the paper’s conclusions.

## 2. Timing Estimation Algorithms

**Figure 1:**
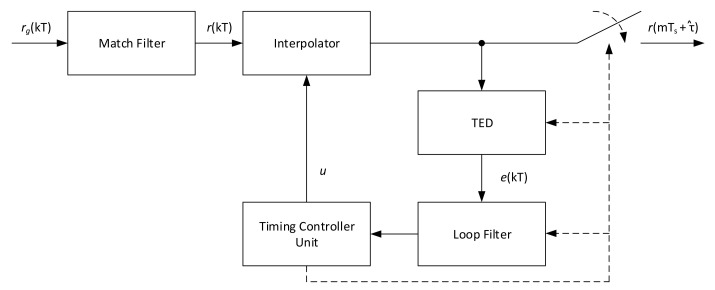
Feedback timing architecture.

**Figure 2:**
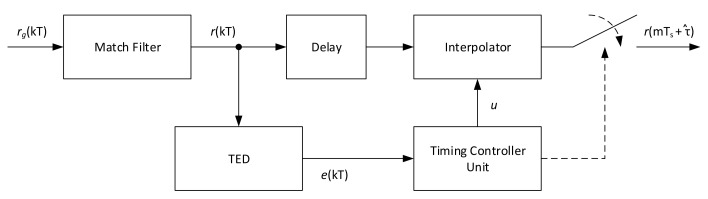
Feedforward timing architecture.

Many timing error detector algorithms have been developed over the year, but no specific evaluation of all these methods is present in literature on the CCSDS 131.2-B-1 standard considering its working $E_{s}/N_{0}$ range and supported constellations. To cover this, a set of feedback and feedforward timing error detectors (TED) has been evaluated. The two classes of detectors are depicted respectively in Figure 1 and Figure 2, respectively. It should be noticed that all these systems are based on asynchronous sampling with respect to the symbol clock, thus interpolation is employed to correctly get the symbol values at the ideal sampling instant.

In the feedback model, the output of the match filter is interpolated according the fractional delay μ, which represents the position of the desired sample between the input stream. Based on these samples and a control signal named strobe, the TED evaluates the timing error to be filtered by the loop-filter. The output of the latter module is then elaborated by the timing controller for the calculation of the next value for μ and strobe, thus closing the loop structure. A complete description of feedback loop structures with interpolation can be found in [14].

For feedforward structures, the output of the match filter is directly used by the TED for the error calculation. The error output is feed to the timing controller to calculate the fractional delay μ in a similar manner with respect to the feedback loop, also creating the sampling instant (strobe). To compensate for the TED estimation processing time, a delay block might be placed on the sample stream line, so the estimation is exactly applied to the samples on which it is evaluated.

For the evaluation of the different algorithms, the received signal after matched filtering is assumed to be in the form:

(1) $$r\left(t\right)=x\left(t-\tau \right)e^{j\left(2\pi \Delta ft+\theta \right)}+\nu \left(t\right)$$

where x(t) is the received complex signal obtained as convolution of symbols and transmitter/receiver Square Root Raised Cosine (SRRC) filters; τ is a time delay in the range ($-T_{s}/2$, $T_{s}/2$] with $T_{s}$ equal to the symbol duration; Δf is the residual frequency offset; ϕ is the received signal phase; and ν(t) is a complex additive white gaussian noise (AWGN) process. The discrete time signal for the digital signal processing chain would be oversampled by *N* with respect to the ideal symbol period $T_{s}$. The notation $r_{I}$ and $r_{Q}$, used in some of the timing error detector formulations for improved clarity, reflect the in-phase and quadrature components of signals, respectively.

### 2.1. Early-Late Timing Error Detector (NDA-FB)

The Early-Late timing recovery algorithm is one of the vary basic ones. It is based on approximations for the derivative part in the Maximum Likelihood (ML) detector.

(2) $$e\left[m\right]=r\left(mT_{s}+{\hat{\tau }}_{m}\right)\;\cdot \;r^{\prime }\left(mT_{s}+{\hat{\tau }}_{m}\right)$$

where the derivative $r^{\prime }$ sample (obtained filtering the received samples with the derivative matched filter) is substituted by a difference between two samples. The Early-Late detector (EL-TED) formulation based on the I and Q rails can be therefore derived as:

(3) $$\begin{array}{cc} e\left[m\right]= & r_{I}\left(mT_{s}+{\hat{\tau }}_{m}\right)\;\cdot \;\left[r_{I}\left(\left(m+1/2\right)T_{s}+{\hat{\tau }}_{m}\right)-r_{I}\left(\left(m-1/2\right)T_{s}+{\hat{\tau }}_{m}\right)\right]+\\ \; & x_{Q}\left(mT_{s}+{\hat{\tau }}_{m}\right)\;\cdot \;\left[r_{Q}\left(\left(m+1/2\right)T_{s}+{\hat{\tau }}_{m}\right)-r_{Q}\left(\left(m-1/2\right)T_{s}+{\hat{\tau }}_{m}\right)\right] \end{array}$$

### 2.2. Gardner Timing Error Detector (NDA-FB)

The Gardner timing recovery algorithm, developed in 1986 by Floyd M. Gardner [4], is one of the most popular feedback TEDs. Gardner timing error detector can be considered as derived from the maximum likelihood principle as demonstrated in [15]. The Gardner TED (GD-TED) requires at least two samples per symbol and knowledge of the previous symbol timing in order to estimate the timing error for the current symbol. The timing error computation based on the I and Q rails is computed as depicted in (4).

(4) $$\begin{array}{cc} e\left[m\right]=r_{I}\left(\left(m-1/2\right)T_{s}+{\hat{\tau }}_{m}\right)\;\cdot \;\left[r_{I}\left(\left(m-1\right)T_{s}+{\hat{\tau }}_{m}\right)-r_{I}\left(mT_{s}+{\hat{\tau }}_{m}\right)\right]+\\ \; & r_{Q}\left(\left(m-1/2\right)T_{s}+\hat{\tau }\right)\;\cdot \;\left[r_{Q}\left(\left(m-1\right)T_{s}+{\hat{\tau }}_{m}\right)-r_{Q}\left(mT_{s}+{\hat{\tau }}_{m}\right)\right] \end{array}$$

### 2.3. Zero-Crossing Timing Error Detector (DD-FB)

Among with non-data aided detectors, the analysis also evaluates the performances of Decision-Directed (DD) timing estimators. Due to the missing information about estimated symbols back from the decoding stages or good hard-decoded values at low Signal to Noise Ratio (SNR), the estimation for the direct-decision is based on a simple sign(x) threshold mechanism for the I and Q rails. The Zero-Crossing TED (ZC-TED) timing error [5] computation based on the I and Q rails is computed as:

(5) $$\begin{array}{cc} e\left[m\right]= & r_{I}\left(\left(m-1/2\right)T_{s}+{\hat{\tau }}_{m}\right)\;\cdot \;\left[{\hat{a}}_{I}\left(m-1\right)-{\hat{a}}_{I}\left(m\right)\right]+\\ \; & r_{Q}\left(\left(m-1/2\right)T_{s}+{\hat{\tau }}_{m}\right)\;\cdot \;\left[{\hat{a}}_{Q}\left(m-1\right)-{\hat{a}}_{Q}\left(m\right)\right] \end{array}$$

where the values for the decision variables are obtained as:

$$\begin{array}{cc} {\hat{a}}_{I}\left(m\right) & =sign\{r_{I}\left(mT_{s}+{\hat{\tau }}_{m}\right)\}\\ {\hat{a}}_{Q}\left(m\right) & =sign\{r_{Q}\left(mT_{s}+{\hat{\tau }}_{m}\right)\} \end{array}$$

### 2.4. Mueller and Muller Timing Error Detector (NDA-FB)

In a similar way to the Zero-Crossing detector, also the Decision-Directed Mueller and Muller (M&M) algorithm, developed in 1976 [6], is evaluated. It is usually very popular in coherent receivers employing BPSK or QPSK because it can provide an ISI free output. Moreover, it can also be used at only one sample per symbol, which is lower than the minimum two sample per symbols required by the other feedback TEDs. The formulation of the MM-TED, based on the I and Q rail values, is computed as:

(6) $$\begin{array}{c} e\left[m\right]={\hat{a}}_{I}\left(m-1\right)\;\cdot \;r_{I}\left(mT_{s}+{\hat{\tau }}_{m}\right)-{\hat{a}}_{I}\left(m\right)\;\cdot \;r_{I}\left(\left(m-1\right)T_{s}+{\hat{\tau }}_{m}\right)+\\ {\hat{a}}_{Q}\left(m-1\right)\;\cdot \;r_{Q}\left(mT_{s}+{\hat{\tau }}_{m}\right)-{\hat{a}}_{Q}\left(m\right)\;\cdot \;r_{Q}\left(\left(m-1\right)T_{s}+{\hat{\tau }}_{m}\right) \end{array}$$

where the values for the decision variables are obtained in the same way as in the Zero-Crossing method, due to the fact that all consideration about constellations and noise levels are valid for this method as well.

### 2.5. Oerder and Meyr Timing Error Detector (NDA-FF)

The Oerder and Meyr [7] is one of the first symbol timing error detector made to be employed in a feedforward structure, yet one of the more powerful algorithms in this class. It is based on practical considerations, such as extracting the frequency component using the square of the input signal and derive its phase from the Discrete Fourier Transform (DFT) coefficient. The equation for the estimator is given as:

(7) $$\begin{array}{c} e\left[m\right]=\frac{1}{2\pi }arg\left\{\sum_{k=mNL_{0}}^{\left(m+1\right)NL_{0}-1}{\left|r\left(k\right)\right|}^{2}e^{-j2\pi k/N}\right\} \end{array}$$

where *N* is the signal oversampling factor with respect to the symbol rate and $L_{0}$ is the estimation length in symbols. In fact, feedforward detectors have formulations that already take into account the effect of the loop-filter employed in feedback structures.

Equivalence between feedback and feedforward system is derived as in (8), where $B_{L}$ is the noise equivalent bandwidth of the feedback loop and $T_{s}$ is the symbol period.

(8) $$B_{L}T_{s}=\frac{1}{2L_{0}}$$

### 2.6. Lee Timing Error Detector (NDA-FF)

Following the idea of feedforward estimators, Lee proposed in 2002 [8] an estimator that works at only two samples per symbols instead of the minimum four symbol required by the Oerder and Meyr. This makes it practically useful in high data-rate transmission schemes where parallelization has to be taken into account. In the latter case, having two samples per symbol instead of four samples per symbol (e.g., Oerder and Meyr) roughly requires about half the complexity.

(9) em=12πarg∑k=mLN+1(m+1)LN|r(k)|2e−jkπ+Rer(k)r*(k−1)e−j(k−0.5)π

## 3. Frequency Estimation Algorithms

For the carrier frequency algorithm part of the paper, delay and multiply, Kay [9], Fitz [10], Luise and Reggiannini [11], Mengali and Morelli [12], O’Shea et al. [13] are considered to provide a broadband spectrum of available performances and implementation complexity for CCSDS 131.2-B-1 satellite telemetry downlink receivers. In particular, it is assumed that the received sampled symbols have the form:

(10) $$r\left(k\right)=x\left(k\right)e^{j\left(2\pi \Delta fkT_{s}+\theta \left(k\right)\right)}+\nu \left(k\right)$$

where $T_{s}$ is symbol period; x(k) is the ideal sampled transmitted signal, Δf represents the frequency error, θ(k) is the phase error and ν(k) the channel noise. All algorithms under assessment are considered for a Data-Aided (DA) estimation approach based on symbol values, which means that information about the received signal is used to remove the effect of modulation. This choice to use symbol-based DA estimation stems from the fact that no information is known about the constellation before a PL-frame is recognized, and signal-level FEDs require this type of information to work properly. In addition to this, high-order constellations require very complex signal-level estimators with a high resource footprint compared to symbol Data-Aided ones. Specifically to the CCSDS 131.2-B-1, the presence of a long frame marker, which is a fixed known sequence of symbols used to announce the beginning of the frame, makes the standard perfect to be used for DA frequency estimation. The first procedure to be performed is then the elimination of modulation, which is computed by multiplying the obtained frame marker sequence by the complex conjugate of the known ideal one:

(11) $$z\left(k\right)=r\left(k\right)\;\cdot \;s^{*}\left(k\right)$$

where r(k) are the received symbols and s(k) the ideal frame marker ones. The different algorithms are then evaluated, where $\hat{f}$ is the normalized frequency estimation with respect to the symbol rate $S_{rate}$, thus $\Delta f=\hat{f}\;\cdot \;S_{rate}$.

### 3.1. Delay and Multiply Estimator

**Figure 3:**
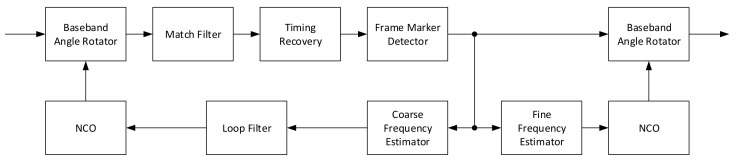
Coarse-fine frequency estimation architecture.

One of the very first estimators to be developed is the so-called delay and multiply method, which only uses the frame marker correlation at distance *D* to perform the calculation. It is known not to be so precise, particularly at low SNR, but it yields unbiased results and the normalized estimation region is $|\hat{f}|<1/2D$. For all those reasons, it is usually employed as a coarse estimator by setting a low *D* value (e.g., 2) in the two stages estimation architecture as the one depicted in Figure 3.

(12) $$\hat{f}=\frac{1}{2\pi D}\;arg\left\{\sum_{k=D}^{L-1}z\left(k\right)z^{*}\left(k-D\right)\right\}$$

### 3.2. Kay Estimator

The Kay estimator developed by Steven Kay in 1987 [9] uses the association between adjacent samples and a w(k) weighting function to improve the precision of the estimates. It is also know from literature that the estimator is easy to implement, outperforming the D&M technique at a high level of SNR. However, it results to be biased for SNR under about 8 dB $E_{s}/N_{0}$, so it is usually not employed for space applications where the operative conditions can be as low as −8 dB $E_{s}/N_{0}$. Kay’s estimator formulation is shown in (13)

(13) $$\hat{f}=\frac{1}{2\pi }\sum_{k=1}^{L-1}w\left(k\right)\;arg\left\{z\left(k\right)z^{*}\left(k-1\right)\right\}$$

where w(k), the weighting function, is equivalent to:

(14) $$w\left(k\right)=\frac{3}{2}\frac{L}{L^{2}-1}\left[1-{\left(\frac{2k-L}{L}\right)}^{2}\right]$$

### 3.3. Fitz Estimator

The Fitz estimator, developed by Michael P. Fitz in 1991, is known for having very accurate and unbiased estimates even at low SNR. However, it also has the narrowest estimation window ($|\hat{f}|<1/2N$) with respect to all the frequency estimators analyzed in this paper. Its formulation is shown in (15) where N is the main design parameter.

(15) $$\hat{f}=\frac{1}{\pi N(N+1)}\sum_{m=1}^{N}\;arg\left\{R(m)\right\}$$

The Fitz estimator exploits, in fact, as other frequency estimators (i.e., Luise and Reggiannini, Mengali and Morelli and O’Shea et al.) the autocorrelation R(m) defined in (16). The value of N in the range N∈[2;128] for CCSDS 131.2-B-1 indicates then the number of autocorrelations that make up the summation. The value of N is crucial in the design of the estimator since it influences both the estimation window (the higher *N*, the narrower the window) and the frequency RMS error (the higher *N*, the lower the error).

(16) $$R\left(m\right)=\frac{1}{L-m}\sum_{k=m}^{L-1}z\left(k\right)z^{*}\left(k-m\right)$$

### 3.4. Luise and Reggiannini Estimator

The Luise and Reggiannini estimator is one of the most widely used estimators in receiver architectures due to the fact that it guarantees a fairly wide estimation range ($|\hat{f}|<1/\left(N+1\right)$) while providing low RMS error. Compared to similar autocorrelation-based estimators, its implementation is simpler because it can be implemented using an Finite Impulse Response (FIR) filter-like architecture. For those reasons it is usually implemented as feedforward fine estimator in a coarse-fine architecture in high data-rates receiver, where the resources required by more complex algorithms (i.e., Fitz, which has several parallel accumulations and angle calculations) are very high. The coarse-fine architecture already shown in Figure 3 allows a wide range estimator, namely coarse, to bring the residual error in the range of the finer one that is able to follow the error with much better accuracy. Due to the limited $|\hat{f}|$ estimation window, when tracking is being performed (i.e., the fine feedforward estimator of Figure 3 is working) in environment with high Doppler-rate, additional logic is needed to correctly unwrap the estimates and track when they fall out of $|\hat{f}|$ [16]. This additional logic, similarly to what has been said in feedforward timing detectors, allows a limited estimation window to be enlarged so no catastrophic behavior happens (e.g., a completely wrong frequency correction value).

(17) $$\hat{f}=\frac{1}{\pi (N+1)}\;arg\left\{\sum_{m=1}^{N}R\left(m\right)\right\}$$

### 3.5. Mengali and Morelli Estimator

One of the most widely used algorithms in receiver architectures, when a broad range of estimates is needed (close to $|\hat{f}|<1/2$), is the Mengali and Morelli [12]. In fact, unlike Fitz and L&R, its estimation range does not depend on the number of autocorrelation samples. The *N* parameter affects the RMS error only, which decreases as *N* increases. Along with RMS, it is also possible to see from its formulation (18) that when *N* increases, the implementation complexity increases too.

(18) $$\hat{f}=\frac{1}{2\pi }\sum_{m=1}^{N}w\left(m\right)\;arg\left\{R\left(m\right)R^{*}\left(m-1\right)\right\}$$

where the smoothing function w(m) is equivalent to:

(19) $$w\left(m\right)=\frac{3((L-m)(L-m-1)-N(L-N))}{N(4N^{2}-6NL+3L^{2}-1}$$

**Figure 4:**
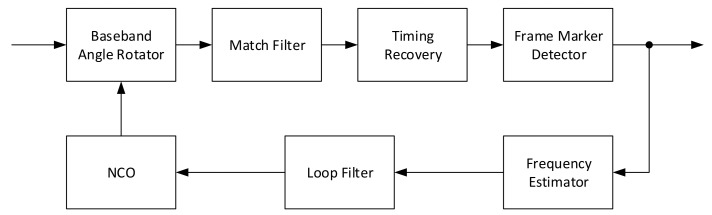
Single loopback frequency estimation architecture.

From a complexity point of view, a direct implementation of the algorithm is quite heavy due to the to the autocorrelation multiplication. However, the Equation (18) can be easily transformed into (20) considering how autocorrelation works.

(20) $$\hat{f}=\frac{1}{2\pi }\sum_{m=1}^{N}w\left(m\right){\left[arg\left\{R(m)\right\}-\;arg\left\{R(m-1)\right\}\right]}_{2\pi }$$

where ${\left[x\right]}_{2\pi }$ represent the modulo 2π. With this transformation, some $arg\left\{R(m)\right\}$ can be reused in a serial architecture, which increases by one the number of *arg* operations, but highly decreases the *arg* operation datapath. Due to its wide estimation range, independent on *N*, it is one of the usual candidate to be implemented in architectures that feature a single loopback frequency estimation (e.g., architecture in Figure 4).

### 3.6. O’Shea et al. Estimator

Finally, a variant of the M&M developed by O’Shea et al. [13] is evaluated. The estimator simply takes out the angle calculation within (18), thus reducing the number of angle calculation to be performed. However, it also complicates the autocorrelation multiplications, which cannot be simplified as in M&M. Depending on the implementation architecture, this may result in lower or increased resources occupation. From the RMS error performance point of view, the estimator shows a loss with respect to M&M due to the fact that the argument of the weighted sum is used (same coefficients as M&M), but the estimation range borders are more consistent.

(21) $$\hat{f}=\frac{1}{2\pi }\;arg\left\{\sum_{m=1}^{N}w\left(m\right)R\left(m\right)R^{*}\left(m-1\right)\right\}$$

## 4. Timing Detectors Performance and Trade-Off

In this section a review all the timing estimator under analysis, starting from the feedback ones, is done. The feedback estimators were in first place characterized by their S-Curves. The S-Curves were taken considering the input signal as normalized to a reference power level (e.g., 0 dBm), showing the degradation of the estimates when the signal noise level increases. This power level normalization takes into consideration the Digital Signal Processing (DSP) stages and the Analog to Digital Converters (ADC). The latter, in fact, usually have Variable Gain Amplifiers (VGA) before digitization to better exploit the input dynamic, avoiding the saturation of r(t)=x(t)+ν(t). The evaluation was specifically done by considering the ModCod performance table in [17] as a reference for the $E_{s}/N_{0}$ values to be used. In fact, the CCSDS 131.2-B-1 standard defines a list of 27 ModCod with very different coding-rates and constellations to better exploit the channel at different noise levels. Each ModCod has been standardized to optimize the data transfer for a specific noise level range, however there is no restriction for using only a subsets of the list or using a ModCod outside its best performance range.

The values from [17] providing the information of usual operative ranges for the different ModCods are resumed in Table 1 for the different modulations. The evaluation of the timing estimators, however, has been performed on a slightly increased range on both ends to account classical receiver operations. It is in fact usual that a receiver is required to acquire the lock status on timing, frame, frequency, phase before the noise level value where the BER starts to fall from 0.5 to 0. This guarantees that the system is ready to receive data as soon as the noise level goes in the operative range. The extended range on the upper side is instead considered to account that the ModCod change may not be at the ideal point but the system may wait for a slightly reduced noise floor before jumping to a higher ModCod for improved reliability. Moreover, there was no need to test all CCSDS 131.2-B-1 ModCods, because only the shape of the constellation is accountable for the timing recovery performance. Similar constellations (i.e., the four 16-APSKs and the three 32-APSKs) are only considered once due to the fact that they share a very close radius ratio, thus similar performance.

**Figure 5:**
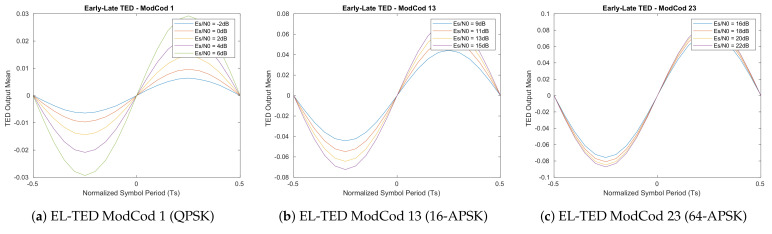
Feedback Early-Late timing estimator S-Curves (500 M symbols).

**Figure 6:**
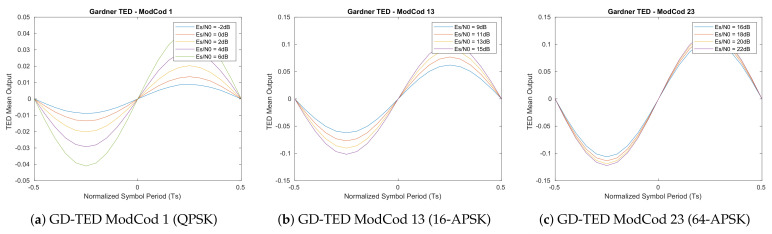
Feedback Gardner timing estimator S-Curves (500 M symbols).

**Figure 7:**
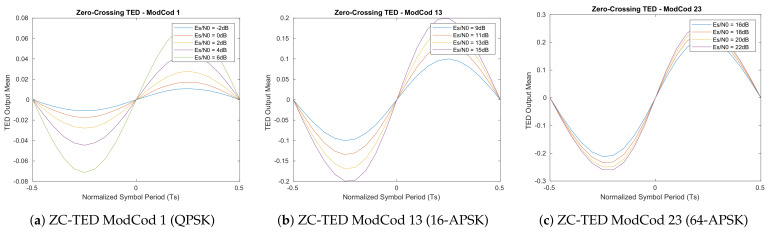
Feedback Zero-Crossing timing estimator S-Curves (500 M symbols).

**Figure 8:**
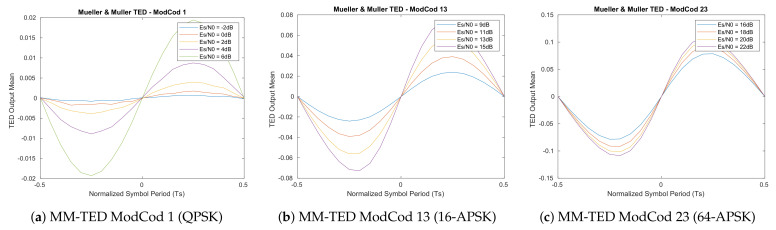
Feedback Mueller and Muller timing estimator S-Curves (500 M symbols).

**Figure 9:**
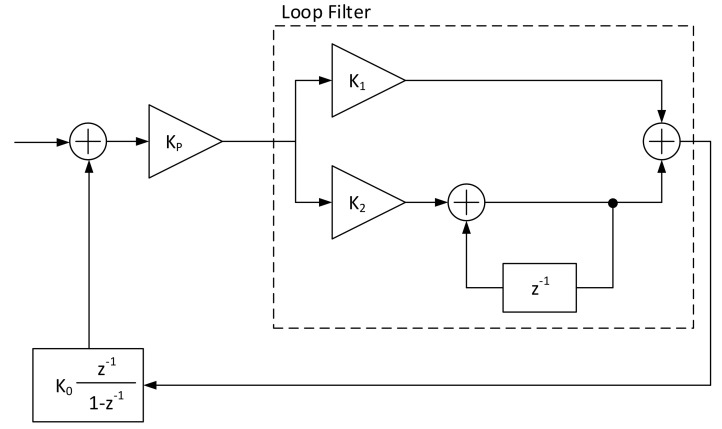
Generic system level view of a second order loop filter.

The resulted S-Curves of this first analysis (Figure 5, Figure 6, Figure 7 and Figure 8) show how the error detector responds to a signal having a normalized delay $\Delta_{T}$ between −0.5 and 0.5 of the symbol timing. These plots are obtained at different levels of $E_{s}/N_{0}$, taking also into account the relative recommended ModCod for that noise level. In particular, the value around $\Delta_{T}=0$ represents the stable point of the system while the ones on $\Delta_{T}=-0.5$ or $\Delta_{T}=0.5$ are the unstable ones. The amplitude of the response determines also the amplitude of the signal going into the loop filter of the loopback structure. In order to obtain a response similar for all the estimators, thus a normalize loop bandwidth, it is therefore important to know the shape and the slope around the stable point. From the S-Curves, the main parameter to extract is the TEDs gain (*Kp*) to be used for the closed feedback simulations as the slope of S-Curves for τ=0. Due to the normalized power at the input of the receiver, a rational choice is to use the minimum slope among the possible noise cases. A second order loop filter, as the one depicted in the generic locked loop of Figure 9, was employed in the analysis to compensate both time shift and compression/dilation effects on symbols due to mismatch between the transmitter and receiver clocks or Doppler.

**Figure 10:**
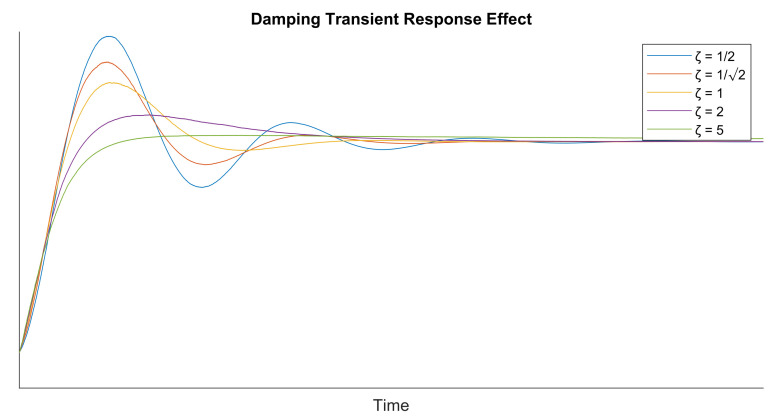
Damping effect on transient response.

The other parameter to characterize the feedback structure is the value of the NCO gain that can be set to $K_{0}=-1$. The user defined parameters on the other side are the damping factor ζ; the equivalent loop bandwidth normalized to the symbol rate $B_{L}T_{s}$; and the signal oversampling factor *N* w.r.t. the symbol rate. The damping factor effect on the time response of a feedback loop is depicted in Figure 10.

From all these settings, exploiting (22) and (23), it is possible to obtain the values for the proportional gain (*K*1) and the integrative gain (*K*2) of the loop filter.

(22) $$K_{p}K_{0}K_{1}=\frac{4\zeta \frac{B_{L}T_{s}}{N\left(\zeta +\frac{1}{4\zeta }\right)}}{1+2\zeta \frac{B_{L}T_{s}}{N\left(\zeta +\frac{1}{4\zeta }\right)}+{\left(\frac{B_{L}T_{s}}{N\left(\zeta +\frac{1}{4\zeta }\right)}\right)}^{2}}$$

(23) $$K_{p}K_{0}K_{2}=\frac{4{\left(\frac{B_{L}T_{s}}{N\left(\zeta +\frac{1}{4\zeta }\right)}\right)}^{2}}{1+2\zeta \frac{B_{L}T_{s}}{N\left(\zeta +\frac{1}{4\zeta }\right)}+{\left(\frac{B_{L}T_{s}}{N\left(\zeta +\frac{1}{4\zeta }\right)}\right)}^{2}}$$

For the evaluation of the jitter, all the feedback and feedforward systems have been evaluated using $B_{L}T_{s}=10^{-4}$, which is a common value for LEO space applications. The equivalent $L_{0}$ value for feedforward estimators is $L_{0}=5000$. For what concerns the detector gain *Kp* to be used in simulations for feedback TEDs, a reasonable choice is to use the one for the worst condition (i.e., slowest S-Curve slope for τ=0). This also comes with the idea that in principle it is also not possible to know the operative ModCod or noise level before symbol recovery and frame synchronization. The ModCod value is in fact embedded in the PL-Frame structure, and its value may change based on channel conditions when using ACM.

The jitter values were evaluated under the condition of having $B_{L}T_{s}=10^{-4}$ and damping factor ζ=1, and an SRRC roll-off factor α=0.25. For all the feedback-based TED, the estimations were done using N=4; for the Oeder and Meyr feedforward estimator the minimum value of N=4 is retained; and for Lee TED the signal oversampling was set to N=2. The jitter values were then compared to the Modified Cramer–Rao Bound (MCRB) defined in [18] as a reference to performances limit (note *MCRB* < *CRB*). The expression for MCRB is shown in (24) in the case of having a Root Raised Cosine (RRC) global filter response, where α is the roll-off factor.

(24) $$MCRB\left(\tau \right)=\frac{B_{L}T_{s}}{4\pi^{2}\xi }\frac{T_{s}^{2}}{E_{s}/N_{0}}$$

$$\xi =\frac{\int_{-\infty }^{\infty }T_{s}^{2}f^{2}{\left|G\left(f\right)\right|}^{2}df}{\int_{-\infty }^{\infty }{\left|G\left(f\right)\right|}^{2}df}=\frac{1+3\alpha^{2}-24{\left(\alpha /\pi \right)}^{2}}{12}$$

**Figure 11:**
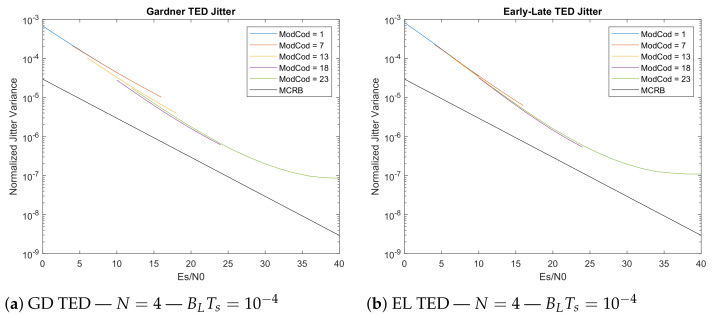
Gardner TED and Early-Late TED jitter evaluation (5 × 10^8^ transmitted symbols).

The main characteristic of jitter simulations is they show that the feedforward estimators do perform better than others when evaluated at the same value of $B_{L}T_{s}$. Comparing the feedback ones, the Gardner TED in Figure 11a and Early-Late TED of Figure 11b show very similar performances. In low SNR conditions where the standard suggests to use QPSK modulation, the Gardner TED shows 17.7% reduced jitter w.r.t. to the Early-Late, while at high SNRs (e.g., $E_{s}/N_{0}$ = 26 dB with 64-APSK) it is the Early-Late that shows 6.4% less jitter w.r.t. to the Gardner TED.

**Figure 12:**
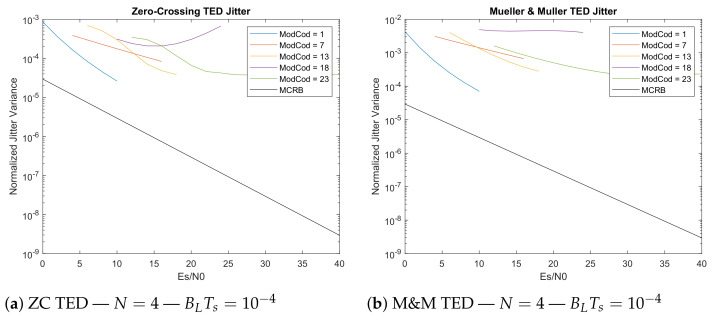
Zero-Crossing TED and M&M TED jitter evaluation (5 × 10^8^ transmitted symbols).

Regarding the Decision-Directed estimators, it is possible to see from the Figure 12a,b that the absence of information about the constellation, which forces the decision to be performed in a simplified way, decreases the performances. The jitter is therefore much higher compared to NDA estimators, and it should also be noted that DDs require prior compensation of residual frequency and phase offsets to work properly. This makes it very difficult to implement them in systems that support multiple constellations such as the CCSDS 131.2-B-1 under analysis. In fact, it is not possible to know the constellation order before decoding the Frame Descriptor, and consequently it is not possible to perform any of the classical power-law algorithms needed to remove the modulation effect noise on the frequency and phase estimates. For this reason, DD estimators are not considered for a possible implementation on the receiver architecture.

**Figure 13:**
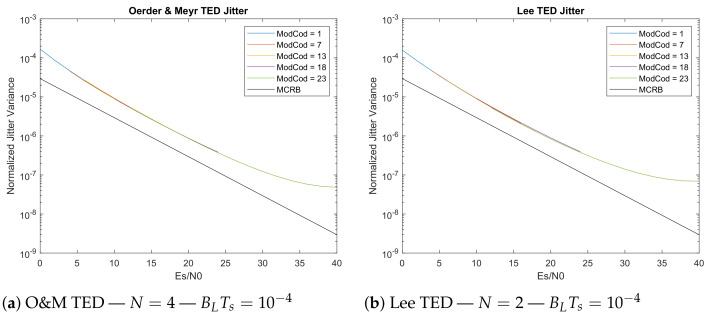
Oerder and Meyr TED and Lee TED jitter evaluation (5 × 10^8^ transmitted symbols).

Finally the feedforward systems are the one providing the best performance (i.e., Figure 13) at the same $B_{L}T_{s}$. The Oerder and Meyr technique shows about x6 reduced jitter when compared to the Gardner and Early-Late feedback loops at low SNRs. At high SNR (i.e., $E_{s}/N_{0}$ = 26 dB with 64-APSK) this difference is reduced to ×1.69. When compared to the Lee estimator the performances of Oerder and Meyr TED provide almost the same jitter profile up to Es/N0 = 24 dB, while at higher SNRs the O&M converges to a floor level having about 30% reduced jitter w.r.t Lee’s TED. However, it has to be considered that Lee’s estimator runs at oversampling N=2, while O&M is forced to a value equal or higher than 4 (e.g., *N* = 4 in this analysis). This represents a big improvement in high data-rate environments where the parallelization level, thus complexity, linearly depends on the oversampling factor of the receiver more than the algorithmic difference between the two (which are very similar).

Apart from jitter related consideration, it should be also pointed out that feedforward timing error detectors need additional logic to correct the estimates, which are limited to $\tau \in (-0.5\;T_{s};0.5\;T_{s}]$. When error falls out this range, the output may be effected by and error equal to $T_{s}$ with respect to the current sampling basepoint. Structures that aim to compensate this may be based on the cyclic nature of this effect in the presence of ramp-like timing error, or using an unwrapping technique to extend the range. The first one is also based on a feedforward structure, while unwrapping is based on a feedback structure. In the presented receiver architecture, the unwrapping technique is used because it provides finer control. This is due to the fact that it is based on actual real-time estimates and not a long-term approximation of the general nature of these.

## 5. Frequency Estimators Performance and Trade-Off

**Figure 14:**
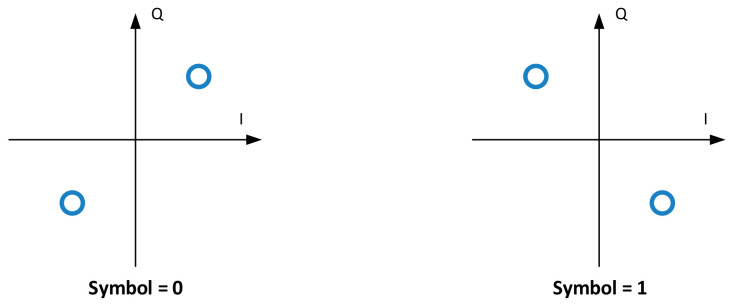
π/2 BPSK frame marker symbols.

All frequency estimators were compared on the specific 256-symbol CCSDS 131.2-B-1 frame marker sequence. The latter symbols are mapped, similar to DVB-S2, onto a π/2 BPSK constellation as shown in Figure 14.

Performance was then evaluated on AWGN channel, where the worst case scenario among all simulations was set to $E_{b}/N_{0}$ @ ModCod = 1 ($E_{s}/N_{0}≊E_{b}/N_{0}-1.4821\;$ [dB]), which is at the operative limit for the standard. In the latter case, receiver could only successfully lock to the frame structure (e.g., timing, frame detection, frequency and phase), because the Bit Error Rate (BER) characteristic is near 0.5 [17], meaning that the system is not able to decode data bits.

**Figure 15:**
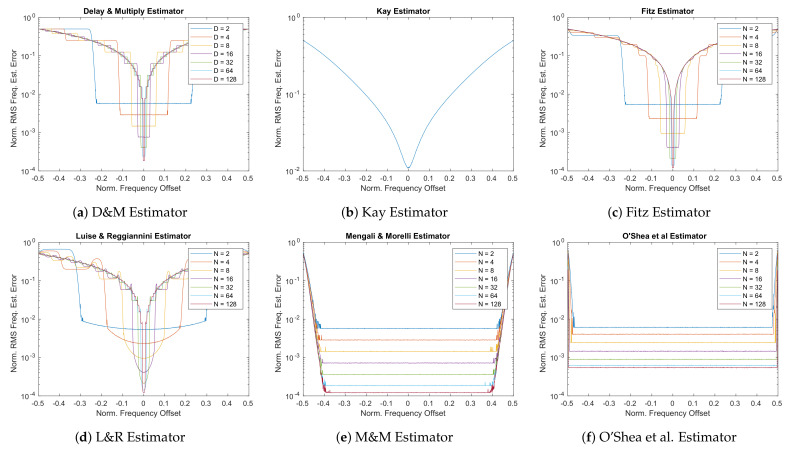
Frequency Estimators Performance, frequency offset sweep, *E_b_*/*N_0_* = −0.52 [dB].

The first set of plots performed to analyze the different frequency estimator ranges shown in Figure 15 are obtained at $E_{b}/N_{0}$ = -0.52 dB ($E_{s}/N_{0}$≊−2 dB). From these graphs, it can be seen that, due to its low precision and narrow estimation range, the performances of the Kay estimator in Figure 15b are not usable for space applications as expected. The O’Shea et al. estimator in Figure 15f, although it has the broadest estimation range, reports a higher RMS frequency error than the other estimators, including the very simple D&M estimator of Figure 15a for the design parameter D=N. For what concerns the Fitz estimator, it shows very similar estimation range w.r.t. the D&M, but the estimates are more accurate (especially at low *N* values).

**Figure 16:**
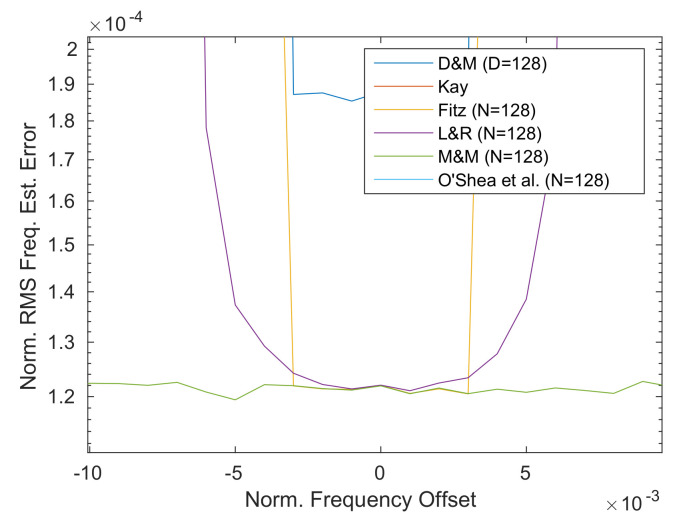
Frequency estimation range details (*D* = 128; *N* = 128).

Comparing then the three most precise estimators, i.e., Fitz, L&R, M&M, it is possible to see that at low values of *N* the difference between the three is very neat. Then, at higher *N* values (e.g., *N* = 64 or *N* = 128) the difference between these estimators tends to reduces and the three estimators converge to very similar results as depicted in Figure 16.

**Figure 17:**
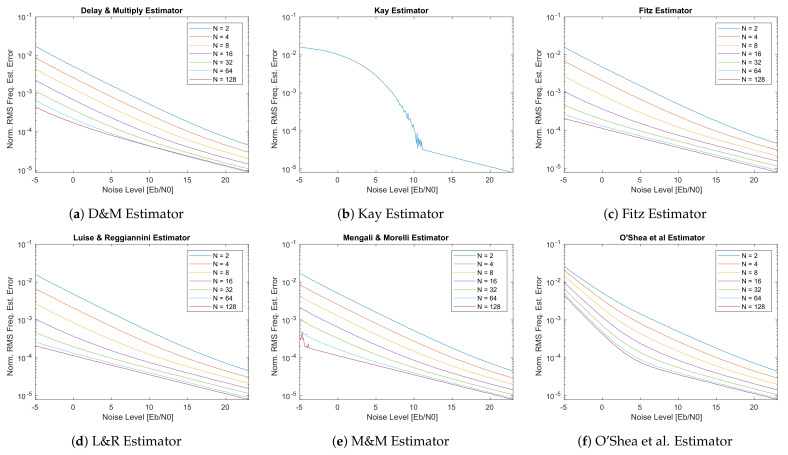
Frequency Estimators Performance, no frequency offset, *E_b_*/*N_0_* sweep.

Finally, one thing to note is that L&R shows a non-perfectly flat estimate within the estimation range, which can be up to 1.5× the lower high-N value (i.e., *N* = 128) as visible in Figure 16. This effect has to be checked when the system is planned to be used also near its estimation edges. As a next step in the analysis to check the performances with different channel conditions, i.e., different values of $E_{b}/N_{0}$, another set of simulations has been performed using a carrier frequency offset Δf=0, while sweeping the noise level $E_{b}/N_{0}$ from −5 dB to 23 dB to simulate the different noise condition in the CCSDS 131.2-B-1 design range. The output of this simulation is shown in Figure 17.

**Figure 18:**
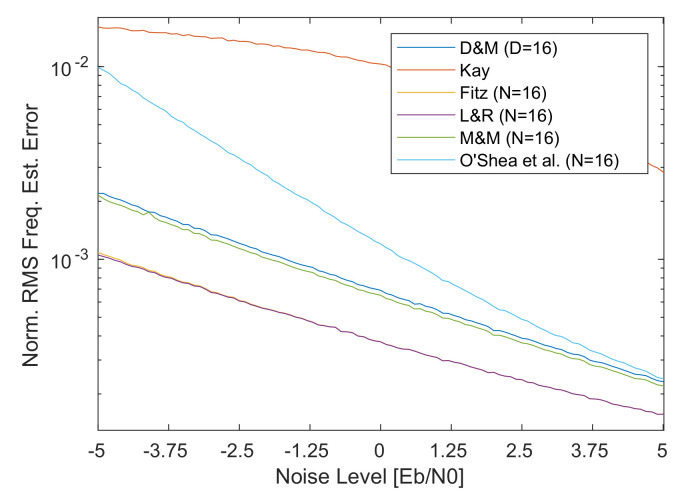
Frequency estimation noise detail (*D* = 16; *N* = 16).

The first thing that comes to the eyes is that, as expected, the Kay estimator in Figure 17b immediately increases the frequency error for low SNRs. This further confirms that not only the previously simulated noise level of $E_{b}/N_{0}$ = −0.52 dB is critical for this estimator, but all the ranges before the 11 dB value. The second thing to note is that the O’Shea et al. in Figure 17f shows a rapid increase in the RMS error for $E_{b}/N_{0}$ lower than 5 dB. This might not be crucial for low values of *N*, but for high values of this tuning parameter the effect becomes more pronounced. As for the delay and multiply algorithm (Figure 17a) it shows good performance, but compared to Fitz and L&R it is characterized by a 2× higher frequency RMS error in the example case of having *N*=16 and very low SNR. Comparing D&M and M&M, it is possible to see that the M&M for low values of N (e.g., *N* = 16) show performances similar to the D&M, while at higher *N* values this difference becomes more pronounced in favor of M&M. Finally, when comparing the Fitz, L&R and M&M, the difference is about 2× between the M&M w.r.t. the Fitz or L&R as visible in Figure 18) for *N* = 16. At high values of *N*, this difference between M&M w.r.t. Fitz or L&R, become less pronounced (i.e., 1.29× at *N* = 64). To compare Fitz and L&R, it is important to recall that from a direct implementation complexity versus performances, the L&R is advantaged by its easier FIR-like implementation.

## 6. System Architecture

**Figure 19:**
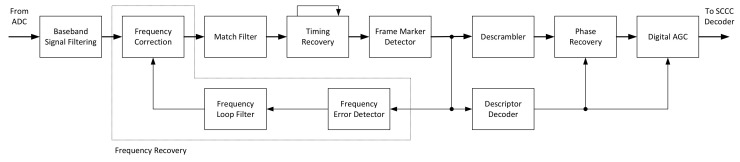
Proposed CCSDS 131.2-B-1 receiver architecture.

All the analyses performed in the paper, along with other architectural choices, did contribute to the realization of the receiver architecture presented in Figure 19. The main architectural choices for the receiver are derived from the fact that it is difficult to support a standard with high order constellations (e.g., 32-APSK and 64-APSK). Moreover, it is extremely difficult when the information about the constellation is unknown before recovering it from the frame header. For such reasons, and the fact that modulation blind NDA frequency and phase provide low accuracy results, the only real choice for the CCSDS 131.2-B-1 is to perform them as DA. This choice implies that the symbol timing needs to be recovered before the frequency/phase offset are estimated. Fortunately, many TEDs in literature (i.e., the presented Early-Late, Gardner, Oerder and Meyr and Lee) are mostly independent of the frequency, phase and modulation. Moreover, also correlation-based frame marker detectors are mainly independent on those variables, making it possible to build high performance receivers for those standards.

The proposed receiver architecture, that embeds all developed modules, features a baseband signal filtering stage that it is able to decimate the incoming signal according to the supported symbol rate. The job is to reduce the sample rate to an integer multiple of the symbol rate (i.e., 4) as required by the symbol timing recovery module. The rate reduction is performed by CIC, FIR and fractional rate converters. The next block in the processing chain is the baseband frequency compensation module that is able to correct the residual frequency by rotating the incoming I/Q stream according to the filtered estimates of the frequency offset. The symbol timing recovery, after a standard two-decimating match FIR filter, recovers the timing information in a feedforward manner. In particular, it implements the Lee estimator using a two parallel implementation. In the related Section 6.1, we demonstrate that through smart choices it is possible to achieve a compact implementation along with high performances. Then, the frame marker detector is in charge of finding the frame start position on the incoming symbol stream using the Choi-Lee L3 method [19]. After frame synchronization, the frequency feedback loop is closed by the frequency error detector based on the frame marker sequence described in Section 6.2. The combination of the frequency correction, frequency estimator and frequency loop filter made up the frequency recovery section. The following modules are then the descrambler to invert the symbol pseudo-randomization performed in the transmitter; the phase recovery on the frame marker with interpolation between pilot symbols to recover the carrier phase; and digital automatic gain control (DAGC) for normalizing the *Es* level normalization before going to the SCCC decoder section.

### 6.1. System Architecture—Timing Recovery

The system features Lee’s estimator to recover the symbol timing information on the received samples. This choice has been made as a trade-off when comparing Garner TED and Lee TED, both using *N* = 2 to reduce the implementation area. In fact, both TEDs are hardware efficient when using *N* = 2, but Lee’s shows an increased performance w.r.t. Gardner. The usage of *N* = 2 is derived from the fact that covering symbol rates from 5 Mbaud to 500 Mbaud requires to perform parallelization, because the operative sample-rate is $N\;\cdot \;S_{rate}$. The latter value for 500 Mbaud falls out of any achievable clock frequency of current FPGA technology, so parallel implementation is needed to reduce the clock frequency while maintaining the sample rate. Choosing *N* = 2 instead of *N* = 4, therefore reduces performance slightly, as visible by comparing Lee and Oerder and Meyr, but takes up about half the resources. For this scope a two parallel system running at 500 MHz, on first tier FPGAs as the Xilinx RFSoC Kintex Ultrascale+, was implemented.

**Figure 20:**
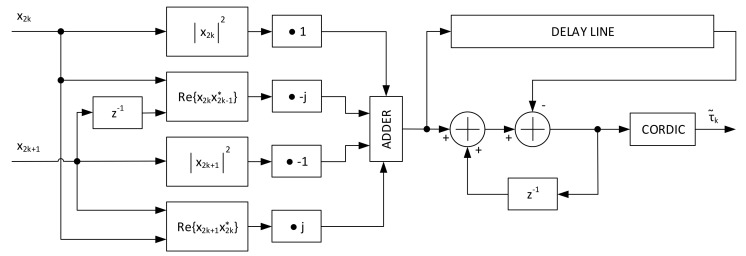
Architecture of the two-parallel Lee timing error detector.

Although it is said in papers [20] that O&M or Lee are computationally heavy for very high data-rates (i.e., 32 Gbaud for optic communications), at the current rates for space communications, if we recall the Formula (9) and look at the structure of FPGAs, we can make efficient use of BRAM blocks to obtain low footprint designs. The idea is to keep track of elements that built up the summation within the angle calculation. With this design choice, it is possible to add the new samples, and remove the old samples avoiding the calculation of all samples summation for each new one. The architecture for a two parallel system employing this structure is depicted in Figure 20. In the first section, the input samples at rate $N\;\cdot \;S_{rate}$ over two parallel lines are feed to the block that calculates the squared modulo and Rer(k)r(k−1)*. The next layer rotates the obtained values by 0, 90°, 180° or 270° degrees according to the formula. It has to be pointed out that the output can be calculated every *N* input samples, so the rotation is fixed in the architecture and the accumulator technique holds its functionality. The accumulated samples are finally processed by a fully pipelined COordinate Rotation DIgital Computer (CORDIC) module to extract the angle information. The 1/2π multiplication can be omitted in hardware since the CORDIC output is already normalized to the output bit dynamic.

**Figure 21:**
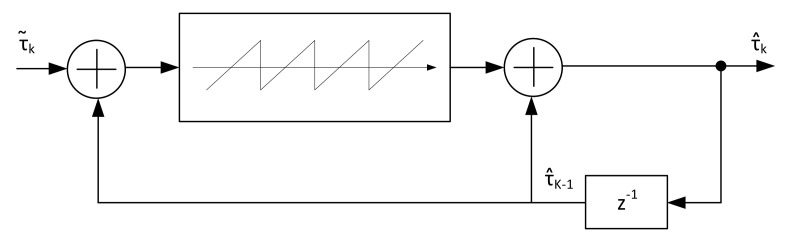
Timing estimates unwrapping module.

After the TED value calculation, unwrapping of the estimates is needed to compensate the reduced estimation range $|\hat{\tau }|<0.5$. This unwrapping technique is executed by the architecture in Figure 21. The system, in the presence of a ramp-like timing offset (e.g., caused by different transmitter/receiver reference clocks) need a normalization to avoid the estimates to grow without control. The information that it is needed to make the system work properly is in fact just the value that wraps to the next/previous symbol, that a system without unwrapping is not able to detect. To avoid this, a normalization is executed when the value oversees 2.5 or −2.5. The output of the unwrapping module embedded in the Timing Controller Unit of Figure 2 provides the intersample position μ and the strobe information for the output sampling by a simple check on $\tilde{\theta }$. The fractional delay μ is then used by an interpolator that exploits a Farrow Structure to implement a third order polynomial interpolation. Implementation results are then provided for the Zynq Ultrascale+ RFSoC FPGA in Table 2 with a maximum clock frequency of 536 MHz. Analyzing the resources occupation, not detailed in the table, all the BRAM blocks are used to store the $L_{0}=5000\;\cdot \;N$ samples. The number of BRAM equal to 5 (up to 5120 locations) instead of 10 derives from the fact that two samples are processed in parallel before being stored in the delay line. For the DSP blocks, 16 are used by the two-parallel third order interpolator, while the other by the estimation algorithm. All the timing loop architecture occupies around 0.68% of the resources onboard the XCZU28DR FPGA.

### 6.2. System Architecture—Frequency Recovery

For what concerns the frequency recovery, a multi-algorithm estimator has been implemented to efficiently cover both high data-rates (e.g., 500 Mbaud) and low data-rates (e.g., 5 Mbaud). The main problem with these very different scenarios is the lock-in phase at system startup and transition to the tracking phase. In the first part, no knowledge about the frequency offset is known, so it is important to have a wide estimator, while in the tracking phase the main objective is to bring the RMS error to the lowest possible values without loosing the lock. If we consider the 5 Mbaud and a Doppler shift of around 1.5 MHz as a worst case scenario, it becomes immediately visible that most the estimators are out of their estimation ranges (even for the lowest values of N) or quite close to the limit. The problem is well reflected on Table 3 where all estimators whose estimation range depends on the *N* parameter are unusable.

The latter analysis reveals that it is important to have a wide range estimator at low symbol rates, while in tracking zone it is possible to use a more narrow, more precise one. This idea is reflected on the implementation where the goal is also set on the reduction of area occupation in the FPGA design. Furthermore, the latter constraint well matches the single feedback loop architecture, so only one estimation block and only one frequency compensation block are needed. However, considering the Formulas (20) and (15), it is also possible to notice that it may be possible to join two different estimators (i.e., M&M and Fits) without significant architectural changes. For the high data-rates, it is possible to consider the 500 Msym/s stream as serial running at least at 500 MHz (in practice a little more to account also for ramp-like timing errors). The latter requirement imposes important architectural choices to ensure that the implementation is pipelined in a way that is able to meet the target clock frequency.

Apart from the implementation related things, it is also important to verify that the system is able to correctly perform the estimates in the presence of Doppler-rate. The latter causes a shift of the received frequencies over time due to the variation in the relative position w.r.t. the ground station. For LEO satellites, the maximum value for the Doppler-rate can be set to 50 KHz/s as a worst case scenario. Since estimates are performed every frame, it is important to calculate the frequency offset that needs to be corrected after the initial Doppler-shift is compensated. The frame rate can be derived using (25) for the CCSDS 131.2-B-1 standard with pilot symbols enabled, which adds 16 symbols every 540 data symbols.

(25) $$\begin{array}{c} R_{FM}=\frac{S_{rate}}{256+64+16\times (8100+16\times 15)}=\frac{S_{rate}}{133760} \end{array}$$

The values are then 37.38 frame/s for the 5 Mbaud case and 3738.04 frame/s for the 500 Mbaud case. The 5 Mbaud case is then clearly the worst scenario because the Doppler-rate causes a higher frequency shift between estimates. Considering 5 Mbaud then, the maximum frequency shift that is a consequence of the Doppler-rate *DR* is:

(26) $$f_{FR}{|}_{max}=\frac{D_{R}}{R_{FM}}=1337.61\;\left[\mathrm{Hz}\right]\;@\;S_{rate}=5\;\mathrm{Mbaud}$$

Since the implementation is focused on a single loopback architecture with a non negligible effect of the Doppler-rate, it is also important to consider the effect introduced by the loop filter for the tracking phase. A Type II Frequency-Locked Loop (FLL) has been considered for the implementation, also taking into consideration the delay caused by the estimation being performed every frame. In this design, the delay can be considered 1 frame long, due to the we can guarantee that all the processing is done before the next frame marker. This fact will be recalled at the end analyzing the number of clock cycles needed by the estimator. The FLL transfer function is therefore:

(27) $$H\left(z\right)=\frac{k_{1}z^{-1}\left(1-z^{-1}+k_{2}z^{-1}\right)}{{\left(1-z^{-1}\right)}^{2}+k_{1}z^{-1}\left(1-z^{-1}+k_{2}z^{-1}\right)}$$

**Figure 22:**
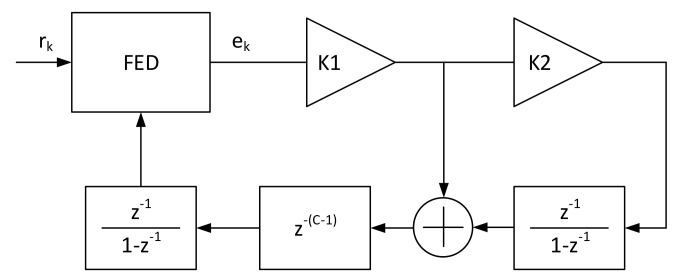
Frequency feedback loop logical scheme.

The need for a Type II FLL as the one of Figure 22 is because of the high residual frequency caused by Doppler-rate in the Type I FLL, and the fact that an interpolating phase recovery is used later in the receiver chain. The latter is able to calculate the phase error between the different pilot blocks, which are spaced by 540 data symbols, and then correct the phase along with a maximum residual frequency offset of:

(28) $$e_{fp-max}=\frac{1}{2\pi }\times \frac{\pi \times S_{rate}}{540}=4.629\;\mathrm{kHz}\;@\;S_{rate}=5\;\mathrm{Mbaud}$$

In this case, a Type I FLL would generate a residual offset (for a frequency ramp) that can be calculated using the final-value theorem for z-transform, as shown in (29).

(29) $$\begin{array}{cc} e_{fp-o} & =\lim_{z\to 1}\frac{D_{R}}{R_{FM}}\frac{1}{\left(1-z^{-1}\right)}E\left(z\right)\\ \; & =\lim_{z\to 1}\frac{D_{R}}{R_{FM}}\frac{1}{\left(1-z^{-1}\right)}[\left(1-H(z)\right){|}_{k_{2}=0}]\\ \; & =\frac{D_{R}}{R_{FM}}\frac{1}{k_{1}}=6.685\;\mathrm{kHz}\;@\;k_{1}=0.2;\;S_{rate}=5\;\mathrm{Mbaud} \end{array}$$

Considering $0<k_{1}<0.25$ for stability reasons, the value of $e_{fp-o}$ is out of the estimation region of the interpolating phase recovery, thus a Type II is needed. A Type II FLL can follow a linear time varying carrier offset with $e_{fp-o}=0$, so only the error jitter is present along with higher order effects (e.g., the sine-like shape of the doppler over time). Under these conditions, an evaluation was carried out on unfiltered FED data for the evaluation of the lock-in between the selected M&M and Fitz algorithms, which share similar equations. Using the common relationship $3\sigma <\frac{1}{4}A_{r}$ that guarantees a high lock-in probability, where *Ar* is the acquisition range, it is possible to see that it holds for most of the N values. In the case of *N*=64 and $S_{rate}$ = 5 Mbaud, we have σ≈930 Hz for the M&M algorithm, while the simulated range of Fitz is around |f|<35,000 Hz. Fitz’s raw estimations show σ≈718 Hz, that simulated in the loop filter with $k_{1}=1/8$ and $k_{2}=1/32$ results in 239 Hz RMS error for the triangular Doppler shape.

**Figure 23:**
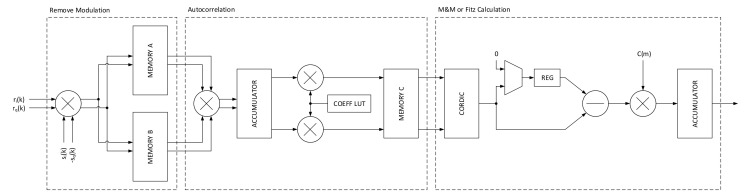
Multi-algorithm (M&M and Fitz) frequency estimator architecture.

With all this information set, we implemented the M&M and Fitz algorithm using *N* = 64. With respect to [21], where only one symbol rate was part of the analysis (8.5 Mbaud), the improved frequency estimator shows a working range of 5 Mbaud to 500 Mbaud with a higher *N* value, meaning also improved accuracy. The architecture of the multi-algorithm estimator, depicted in Figure 23, follows similarly its predecessor with a more balanced pipelining. The first block in Figure 23 has the task of removing the modulation effect on the received symbol sequence by multiplying the incoming symbols related to the frame marker to their ideal counterpart. This concept, as pointed out in the definition of the algorithms, ideally returns a rotating vector with the normalized frequency equal to the one that the system aims to estimate, plus noise. The procedure is performed by a complex multiplication, that however can be resolved to a simple sum if we consider that the frame marker reference symbols may be represented as 1+1j, 1−1j, −1+1j or −1−1j. The obtained z(k) values are then stored in two separate memories to provide efficient parallel access of different z(k) by the autocorrelator module. The second block takes two z(k) each clock cycle and performs all the sample autocorrelation up to the maximum order N required by the implementation. The use of two memory modules makes it possible to calculate one element of the autocorrelation each clock cycle. The autocorrelation module is composed by a fully pipelined complex multiplier for $z\left(k\right)z^{*}\left(k-m\right)$, a complex data accumulator and two 1/(L−m) coefficient multipliers. The output values are again stored into another memory to provide a convenient access for the last module. The last section is the algorithm specific module that processes the different autocorrelations according to the M&M and Fitz estimators. Since the two estimators share similar a similar construction that performs the angle calculation on the autocorrelation values, the first block executes the angle calculation by means of CORDIC. After that, the two estimators differ from one another because the M&M performs a subtraction module 2*pi*, while Fitz goes straight to the weighting multiplier. To accommodate both, a multiplexed is used to conditionally subtract the previous argR(m−1) or zero, thus bypassing the subtraction. The weighting function can select two set of coefficients, one for the M&M and one for Fitz and finally the values are accumulated according (15) or (20). Implementation results for the improved multi-algorithm frequency recovery are then provided in Table 4 for the Zynq Ultrascale+ XCZU28DR RFSoC FPGA. Unlike in [21] the estimation also takes into account the feedback loop filter, an NCO implemented using a coarse-fine approach [22] for the frequency generation and the baseband frequency correction module. The functional representation of the NCO is depicted in Figure 22 by the integrator at the input of the FED module. Analyzing the occupation, the DSP blocks are mainly occupied by the four-parallel NCO and related four-parallel baseband frequency correction modules with 12 and 12, respectively. The BRAM blocks are all used by the frequency estimator module, while the maximum clock frequency is 544 MHz and the processing time 25,196 clock cycles.

## 7. Conclusions

In this paper, we presented the performances of various timing error detector and carrier frequency estimators for the CCSDS 131.2-B-1 satellite downlink standard. A characterization has been performed taking into account the different scenarios derived from both low data-rates (e.g., 5 Mbaud) and high data-rates (e.g., 500 Mbaud) in an LEO environment. The different timing and frequency detectors have been evaluated in terms of jitter and estimation ranges with specific reminders about the possible architectural choices. Finally a receiver architecture was proposed embedding a novel lightweight multi-algorithm frequency loop using M&M and Fitz, while the timing loop exploits a two-parallel Lee feedforward algorithm in a smart way to reduce implementation area.

## Figures and Tables

**Table 1:** Minimum operative *E_s_*/*N_0_* ranges extracted from [17] for timing evaluations.

Constellation (ModCods)	Min. $E_{s}/N_{0}$	Max. $E_{s}/N_{0}$
QPSK (ModCod 1-6)	−0.28 dB	5.84 dB
8PSK (ModCod 7-12)	4.29 dB	11.87 dB
16-APSK (ModCod 13-17)	9.25 dB	14.85 dB
32-APSK (ModCod 18-22)	13.22 dB	18.37 dB
64-APSK (ModCod 23-27)	16.48 dB	21.42 dB

**Table 2:** Timing Recovery Implementation—Zynq Ultrascale+ RFSoC XCZU28DR FPGA.

	LUTs	FFs	BRAMs	DSPs
Absolute	5202	4851	5	21
Percentage	1.223%	0.571%	0.463%	0.491%

**Table 3:** Carrier frequency acquisition for *E_s_*/*N_0_* = −0.52 dB and S_rate_ = 5 Mbaud.

Algorithm	Max. Parameter	RMS Frequency Error
D&M	D = 2	2,500,500 Hz
Fitz	N = 2	1,665,000 Hz
L&R	N = 2	239,050 Hz
M&M	N = 128	705 Hz
O’Shea et al.	N = 128	2726 Hz

**Table 4:** Frequency recovery implementation—Zynq Ultrascale+ RFSoC XCZU28DR FPGA.

	LUTs	FFs	BRAMs	DSPs
Absolute	1723	1511	2.5	32
Percentage	0.406%	0.178%	0.231%	0.720%
